# Comparison of osteopromoting ability of human tooth powder with the demineralized freeze-dried bone allograft, a bovine xenograft, and a synthetic graft: An in vitro study

**DOI:** 10.34172/japid.2020.005

**Published:** 2020-04-26

**Authors:** Mahdi Kadkhodazadeh, Alireza Fathiazar, Zahra Yadegari, Reza Amid

**Affiliations:** ^1^Dental Research Center, Research Institute of Dental Sciences, Dental School, Shahid Beheshti University of Medical Sciences, Tehran, Iran; ^2^Department of Periodontology, Faculty of Dentistry, Ardabil University of Medical Sciences, Ardabil, Iran; ^3^Department of Dental Biomaterials, Dental School, Shahid Beheshti University of Medical Sciences, Tehran, Iran; ^4^Department of Periodontics, Dental School, Shahid Beheshti University of Medical Sciences, Tehran, Iran

**Keywords:** Allografts, Autografts, Bone regeneration, Bone substitutes, Xenografts

## Abstract

**Background:**

The present study aimed to evaluate the osteopromoting ability of human tooth powder and compare it to a bovine xenograft, a synthetic material, and the DFDBA allograft.

**Methods:**

In this in vitro study, 30 teeth without caries, inflammation, and infection, which had been extracted for orthodontic reasons, were collected. The crowns were removed, pulpectomy was carried out, and the samples were ground to a powder with particles <500 µm. Osteoblast-like cells of MG-63 were cultured with the tooth powder, Cerabone, DFDBA, and Osteon II. Cell proliferation was assessed by the MTT assay at 24- and 72-hour intervals. The alizarin red test was carried out after three and five days. The alkaline phosphatase level was measured after 24, 48, and 72 hours to assess the osteoblastic activity. The results were analyzed with one-way ANOVA.

**Results:**

According to the MTT assay, all the materials exhibited a higher proliferation rate than the control group in 24 hours. In 72 hours, DFDBA had the lowest cell proliferation rate at concentrations of 40 and 80 mg/mL. DFDBA and the positive control group were able to create calcified nodules by the alizarin red test. At the 48- and 72-hour intervals, DFDBA had the lowest alkaline phosphatase activity at a concentration of 40 mg/mL. At the 72-hour interval, bovine xenograft had the highest alkaline phosphatase level, followed by the synthetic material and tooth powder.

**Conclusion:**

The tooth powder was able to increase cell proliferation in comparison with the bovine xenograft, the synthetic graft, and the DFDBA. However, its osteopromoting ability was less than that of the osteogenic materials.

## Introduction

Alveolar bone defects are usually treated with autografts, allografts, xenografts, or alloplasts. Autografts are the gold standard for bone grafting; however, they have some complications, such as the need for a second surgery.^1^ The use of bone substitutes, such as allografts and xenografts, has a low possibility of tissue damage; however, they reduce the time of surgery.^2^

A few researchers have tried to use tooth derivatives as graft material for the treatment of bone defects.^3,4^ Tooth and bone are very similar regarding their mineral and organic structures.^4^ The tooth dentin is an excellent source of growth factors and bioactive molecules, such as bone morphogenic proteins (BMPs), transforming growth factor-beta-1 (TGF-β1), and insulin-like growth factor (IGFs).^5^ Collagen type I is the most common extracellular matrix protein component in the dentin and bone. The cross-linked structure of the collagen creates a network which allows the deposition of mineralized crystals.^6^

There is no doubt that the teeth which are extracted every day and thrown away are excellent sources of precious biomolecules and growth factors.^7^ It is crucial to find an easy and inexpensive way to use these precious tooth derivatives in dentistry.^8,9^ Thus, this study aimed to evaluate the osteopromoting ability of powdered human tooth and compare it with a bovine xenograft (Cerabone, Botiss Co, Germany), a synthetic material (Osteon II, Genoss Co, Korea) and DFDBA allograft (Cenobone, Kish, Iran) in an in vitro environment of MG-63 cell line.

## Methods

Thirty intact teeth (without caries, inflammation, and infection), extracted in Shahid Beheshti Dental school for orthodontic reasons, were collected. The crowns of the teeth were removed with a fissured bur, and the roots were rinsed with normal saline solution for five times, followed by a pulpectomy procedure with endodontic files. The roots were crushed with a porcelain mortar and passed through a filter with holes <500 µm. The powder was sterilized with UV radiation for one hour and added to the cultivation medium of Dulbecco’s Modified Eagle’s Medium (DMEM), followed by incubation for 24 hours. An extract with a concentration of 80 mg/mL was made. The extract was diluted to 40, 20, 10, and 5 mg/mL by adding more DMEM.

A bovine xenograft (Cerabone), a synthetic material with HA-TCP (Osteon II), and the DFDBA allograft were sterilized by UV radiation for an hour. As it was described for the powdered teeth, DMEM was added to these materials and incubated for 24 hours. Then, an extract was prepared at a concentration of 80 mg/mL. The extract was diluted to 40, 20, 10, and 5 mg/mL by adding more DMEM, similar to the tooth powder.

### Preparation of cell suspension

The MG-63 cell line provided by the Pasteur Institute (Tehran, Iran) was preserved in a complete cultivation medium composed of DMEM, 10% fetal bovine serum (Gibco Co, Spain), 100 IU/mL of penicillin (Gibco Co, Spain), and 100 mg/mL of streptomycin (Gibco Co, Spain). The solution was protected in an incubator under 5% CO_2_, 95% moisture, and 37ºC. Several passages were carried out to increase the number of cells to 20000 cells/mL for MTT, alkaline phosphatase, and alizarin red tests.

### MTT assay

Osteogenic materials were added to the MG-63 cell line, and the MTT assay was carried out after 24 and 72 hours. Five wells were allocated to the positive control (containing distilled water, which is cytotoxic for cells) and the other five wells to the negative control (containing complete culture medium). Also, five wells were specified for each concentration of 5, 10, 20, 40, and 80 mg/mL of tooth powder, bovine xenograft, HA-TCP, and DFDBA. After 24 and 72 hours, the culture medium was removed, and the wells were washed with phosphate-buffered saline solution (Gibco Co, Spain). Then, 5 mg/mL of the thiazol blue tetrazolium bromide (Sigma, Germany) was added to each well. The plates were incubated for three hours. Afterward, the solution was removed, and 200 mL of dimethyl sulfoxide (DMSO, Sigma, Germany) was added to solve formosan crystals. The range of color absorption was measured by using an ELISA Reader at a wavelength of 570 nm and a reference filter of 620 nm.

### Alizarin red test

Alizarin red test was carried out after three and five days following the incorporation of osteogenic materials to the plates containing the MG-63 cell line. The osteogenic environment was provided (as the positive control) by adding 10 mM ascorbic acid, 50 mM β-glycerophosphate, and 10 mM dexamethasone to the complete culture medium. Two wells were allocated to the positive and negative control groups. Two wells were considered for each concentration of 5, 10, 20, 40, and 80 mg/mL of tooth powder, bovine xenograft, HA-TCP, and DFDBA.

These osteogenic materials were placed next to the MG-63 cells with 20000 cells/mL. After three and five days, the culture medium was removed, and the wells were washed with phosphate-buffered saline solution (Gibco Co, Spain). Then, 70% ethanol was added to each well for one hour as a fixation material. After removing the ethanol, the cells were washed twice with the PBS solution for five minutes. Then, 2% alizarin red (Idezist, Iran) was added to the cells for 30 minutes. Then, the alizarin red was removed, and each well was washed four times with the PBS solution. In the end, calcified nodules and cells’ shapes were tracked with a light microscope at ×40 and ×100 magnification.

### Alkaline phosphatase test

Alkaline phosphatase test was undertaken after 24, 48, and 72 hours following the addition of osteogenic materials to the plates containing the MG-63 cell line.

Four wells were dedicated to both the positive (osteogenic environment) and negative (DMEM) groups. Four wells were allocated to each concentration of 10, 20, and 40 mg/mL of tooth powder, bovine xenograft, HA-TCP, and DFDBA. An alkaline phosphatase kit (Biovision Co, Germany) was utilized to perform the test. The alkaline phosphatase enzyme is a criterion to measure the osteoblastic activity. After 24, 48, and 72 hours, the solution was removed, and the cells were lazed by adding 80 µL of assay buffer to each well; 50 µLof the substrate was also added, and the plates were kept in a dark room for one hour. Then, 20 µL of stop solution was added. The light absorption of the samples was measured by the ELIZA Reader at a wavelength of 405 nm.

### Statistical analysis

SPSS 21 was used to analyze the results of MTT and alkaline phosphatase tests, using means and standard deviations. One-way ANOVA was used to compare the differences between the groups at α≤0.05.

Microscopic observations were used to examine the alizarin red test for the signs of osteoinductivity in the materials.

## Results

### The MTT assay results

**Figure 1:**
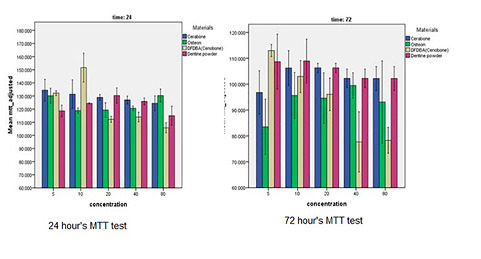
The comparison of MTT assays at 24 hours (left) and 72 hours (right).

The MTT results revealed that none of the materials were cytotoxic after 24 hours of incubation. After 72 hours, Cerabone, Osteon II, and the tooth powder were not cytotoxic, but DFDBA was cytotoxic at high concentrations (40 and 80 mg/mL) (P<0.05) (Table 1 and Figure 1).

**Table 1:** The MTT results for different concentrations of materials

**Material** **mg/mL**	**Cerabone**		**Osteon II**		**DFDBA**		**Tooth Powder**	
	**24 hr MTT%**	**72 hr MTT%**	**24 hr MTT%**	**72 hr MTT%**	**24 hr MTT%**	**72 hr MTT%**	**24 hr MTT%**	**72 hr MTT%**
5	134.42±8.27	96.77±8.39	130.17±5.77	83.52±10.67	132.21±1.93	111.99±2.33	118.66±4.32	108.70±10.62
10	131.39±10.91	106.26±6.72	118.82±1.99	95.62±8.91	151.57±10.93	102.96±6.18	124.41±0.47	108.93±8.44
20	128.91±2.15	106.30±1.77	119.33±5.50	94.62±9.75	112.24±2.36	96.07±6.28	130.35±5.82	106.30±1.77
40	126.96±3.01	102.24±3,58	120.62±1.76	99.49±4.88	113.98±3.65	77.68±11.61	125.85±2.63	102.24±3.58
80	124.45±5.67	102.18±4.64	130.34±4.96	93.11±15.90	105.78±3.91	78.28±4.98	115.11±7.37	102.18±4.64

Values are presented as mean ± standard deviation.

### The alizarin red test results

**Figure 2:**
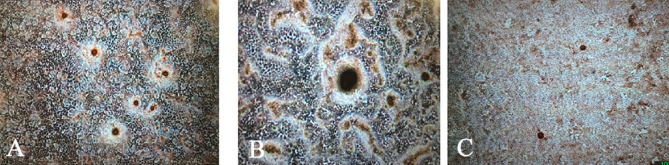
The red round particles are calcified nodules which are stained with alizarin red. A: Positive control with a magnification of ×40. B: DFDBA (40 mg/mL) with a magnification of ×100. C: DFDBA (20 mg/mL) with a magnification of ×40.

Calcified nodules were detected in the positive control and DFDBA groups. There were no signs of calcified materials in any concentrations of Cerabone, Osteon II, and the tooth powder after three and five hours of incubation (Figure 2).

### The alkaline phosphatase test results

**Figure 3:**
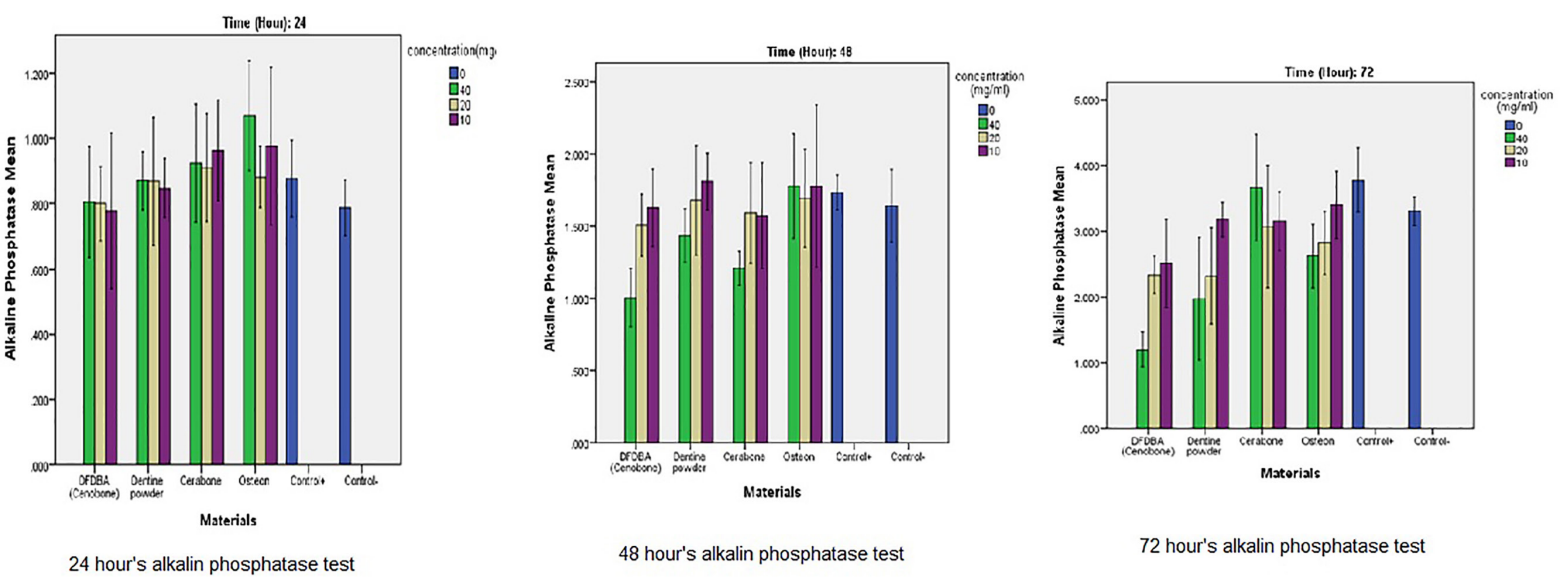
Comparison of alkaline phosphatase levels at 24 hours (A), 48 hours (B), and 72 hours (C).

The results yielded by the ELISA Reader are shown in Table 2. The comparisons between the groups are presented in Figure 3.

**Table 2:** The alkaline phosphatase results for different concentrations

**Material**	**10 mg/mL**			**20 mg/mL**			**40 mg/mL**		
	**24 hours**	**48 hours**	**72 hours**	**24 hours**	**48 hours**	**72 hours**	**24 hours**	**48 hours**	**72 hours**
**Cerabone**	0.96±0.15	1.57±0.36	3.14±0.44	0.91±0.16	1.59±0.34	3.06±0.93	0.92±0.18	1.20±0.11	3.66±0.81
**Osteon II**	0.97±0.24	1.77±0.56	3.40±0.51	0.88±0.09	1.69±0.33	2.82±0.47	1.06±0.16	1.77±0.36	2.62±0.48
**DFDBA**	0.77±0.23	1.62±0.26	2.50±0.67	0.79±0.11	1.50±0.21	2.33±0.28	0.80±0.16	1±0.20	1.19±0.26
**Tooth powder**	0.84±0.08	1.81±0.19	3.17±0.26	0.86±0.19	1.67±0.37	2.31±0.73	0.87±0.08	1.43±0.18	1.97±0.93

Values are presented as mean ± standard deviation.

## Discussion

The 24-hour MTT assay indicated that none of the materials, i.e., the tooth powder, bovine xenograft, HA-TCP, and DFDBA, were cytotoxic, and the proliferation of the cells was more than that in the control group. The notable point was that the tooth powder was able to increase the proliferation of the osteogenic cells.

The 72-hour MTT assay revealed that the tooth powder, bovine xenograft, and HA-TCP were not cytotoxic. However, DFDBA, at 40 and 80 mg/mL concentrations (higher concentrations), was cytotoxic after 72 hours. It might be due to the acidic nature of the DFDBA.

Vaziri et al^10^ assessed the rate of cell proliferation and differentiation of SaOS-2 cells (human osteoblast-like cells) adjacent to DFDBA in 24 and 48 hours. At both intervals, proliferation and viability of the cells were less than those in the control group. In the present study, only at the 72-hour interval, cell proliferation was less than that in the control group. The difference between the results could be due to the different types of cells used in studies. Probably, the MG-63 cell line is more resistant to the acidity of the DFDBA. Generally, as the concentration and duration of the vicinity between DFDBA and the cells increased, cell viability decreased, which could be due to the acidic nature of the DFDBA.

Moharamzadeh et al^11^ evaluated different types of bovine dentin as bone substitutes. They provided a new method in which bovine teeth with open apices were extracted, and after the pulp and PDL were removed, the enamel was removed, too. The teeth were boiled in distilled water for two hours and placed in isopropanol; therefore, the soft tissue was removed completely. Afterward, the material was dried at 100ºC. The biocompatibility was assessed in vitro on human gingival fibroblasts by alamarBlue^TM^. Dentin powder was paced near cells for 72 hours. The results showed that the viability of the cells increased as compared with the control group. The present study also demonstrated that dentin powder could increase cell viability on the MG-63 cells. It should be noted that the dentin powder used was unprocessed human dentin, while this study had used processed bovine dentin.

Vaziri et al^10^ used an alizarin red test to assess the mineralization activity of DFDBA. At a concentration of 16 mg/mL, all the DFDBA types were able to produce calcified nodules after five days, but at a concentration of 8 mg/mL, none of the DFDBAs produced the calcified nodules. They showed that the mineralization was dose-dependent. In the present study, at all the concentrations of DFDBA, calcified nodules were produced. Probably MG-63 cell line can produce calcified nodules easier than the SaOS-2 line.

The alkaline phosphatase test showed no significant difference between the materials at the 24-hour interval. Probably, 24 hours was not enough for significant changes between the groups. At the 72-hour interval, with the concentrations of 10 and 20 mg/mL, no significant difference was detected, but 40-mg/mL DFDBA and tooth powder showed poor results. The bovine xenograft and the synthetic bone substitute showed the highest osteoinductive activity. At the 24- and 48-hour intervals, the tooth powder yielded results comparable to the positive control group, but at the 72-hour interval, its osteoinductive activity decreased, which might be because the teeth were unprocessed.

Kasten et al^12^ evaluated the effect of microporosity of β-tricalcium phosphate on osteogenic differentiation of mesenchymal stem cells. Tests were conducted at 1-, 7-, and 21-day intervals, and the amounts of protein production and alkaline phosphatase were evaluated. In all the groups, TCP was able to increase the alkaline phosphatase levels in 21 hours. In the present study, Osteon II, with 70% B-TCP, increased the alkaline phosphatase levels after 24, 48, and 72 hours. It seems that B-TCP might act as a stimulant to increase alkaline phosphatase levels and osteoblastic activity.

Ayoubian et al^13^ evaluated the biocompatibility and differentiation of SaOS-2 cells near Cerasorb, Osteon, Tutudent, and Bio-Oss. Cell proliferation and biocompatibility were assessed by the MTT assay after 15 days. All the materials showed a lower proliferation rate than the control group, but the alkaline phosphatase test showed more osteoblastic activity than the control group after 15 days. In the present study, Osteon II and Cerabone (a bovine xenograft) gave rise to a higher cell proliferation rate in the MTT assay after 24 hours compared to the control group. Consistent with Ayoubian’s study, the present research revealed that the alkaline phosphatase level in OsteonII and Cerabone was higher in comparison with the control group.

In a recent in vitro study conducted by Tanwatana et al,^14^ several processes, such as thermal and chemical deproteinization of tooth matrix, demonstrated favorable proliferation and attachment of osteoblasts. They concluded that the demineralized and deproteinized tooth matrix is a suitable scaffold for osteoblasts. Consistent with this study, the present study demonstrated suitable properties for the tooth powder; however, the teeth did not undergo any chemical or thermal processes.

The use of autogenous tooth as a bone substitute has been shown in several case-report clinical studies,^15,16^ but more research is necessary to find the best and inexpensive processing method to produce a tooth-derived bone material.^17^

## Conclusion

Tooth powder was able to increase osteogenic cell proliferation in comparison with the bovine xenograft, the synthetic graft, and the DFDBA. However, its osteopromoting ability was less than the osteogenic materials. Further research is necessary to reach a valid conclusion.

## Authors’ Contributions

MK and AF: Conceptualization, AF: Formal Analysis, ZY: Investigation, MK and ZY: Methodology, MK: Project Administration, AF and RA: Writing – Original Draft, AF and RA: Writing – Review & Editing. All authors have read and approved the final manuscript.

## Ethics Approval

The study was conducted in an in-vitro environment and no humans participated in the study. The study did not have any ethical registrations.

## Competing of Interests

The authors declare that there were no competing interests between the authors.
